# Human Obesity Induces Dysfunction and Early Senescence in Adipose Tissue-Derived Mesenchymal Stromal/Stem Cells

**DOI:** 10.3389/fcell.2020.00197

**Published:** 2020-03-26

**Authors:** Sabena M. Conley, LaTonya J. Hickson, Todd A. Kellogg, Travis McKenzie, Julie K. Heimbach, Timucin Taner, Hui Tang, Kyra L. Jordan, Ishran M. Saadiq, John R. Woollard, Busra Isik, Mohsen Afarideh, Tamar Tchkonia, James L. Kirkland, Lilach O. Lerman

**Affiliations:** ^1^Division of Nephrology and Hypertension, Department of Medicine, Mayo Clinic, Rochester, MN, United States; ^2^Division of Geriatric Medicine and Gerontology, Department of Medicine, Mayo Clinic, Rochester, MN, United States; ^3^Department of Surgery, Mayo Clinic, Rochester, MN, United States; ^4^Department of Immunology, Mayo Clinic, Rochester, MN, United States; ^5^Robert and Arlene Kogod Center on Aging, Mayo Clinic, Rochester, MN, United States

**Keywords:** obesity, mesenchymal stem cells, cellular senescence, adipose tissue, cellular dysfunction

## Abstract

**Background:**

Chronic inflammatory conditions like obesity may adversely impact the biological functions underlying the regenerative potential of mesenchymal stromal/stem cells (MSC). Obesity can impair MSC function by inducing cellular senescence, a growth-arrest program that transitions cells to a pro-inflammatory state. However, the effect of obesity on adipose tissue-derived MSC in human subjects remains unclear. We tested the hypothesis that obesity induces senescence and dysfunction in human MSC.

**Methods:**

MSC were harvested from abdominal subcutaneous fat collected from obese and age-matched non-obese subjects (*n* = 40) during bariatric or kidney donation surgeries, respectively. MSC were characterized, their migration and proliferation assessed, and cellular senescence evaluated by gene expression of cell-cycle arrest and senescence-associated secretory phenotype markers. *In vitro* studies tested MSC effect on injured human umbilical vein endothelial cells (HUVEC) function.

**Results:**

Mean age was 59 ± 8 years, 66% were females. Obese subjects had higher body-mass index (BMI) than non-obese. MSC from obese subjects exhibited lower proliferative capacities than non-obese-MSC, suggesting decreased function, whereas their migration remained unchanged. Senescent cell burden and phenotype, manifested as *p16*, *p53*, *IL-6*, and *MCP-1* gene expression, were significantly upregulated in obese subjects’ MSC. BMI correlated directly with expression of *p16*, *p21*, and *IL-6*. Furthermore, co-incubation with non-obese, but not with obese-MSC, restored VEGF expression and tube formation that were blunted in injured HUVEC.

**Conclusion:**

Human obesity triggers an early senescence program in adipose tissue-derived MSC. Thus, obesity-induced cellular injury may alter efficacy of this endogenous repair system and hamper the feasibility of autologous transplantation in obese individuals.

## Introduction

As the epidemic of obesity continues to escalate globally, its prevalence is projected to dramatically increase in the United States, Mexico, and England with 47, 39, and 35% of the population being obese by 2030, respectively (Hruby and Hu, 2015; Hales et al., 2018). Currently, obesity is ranked as the leading cause of preventable disease and mortality in the United States, surpassing smoking (Hennekens and Andreotti, 2013). Furthermore, obesity is the chief contributing factor in the development and progression of comorbid complications, such as diabetes, chronic kidney disease (CKD), and cardiovascular events (Choung et al., 2019). Standard classification defines obesity as a body-mass index (BMI) greater than 30 kg/m^2^, whereas a BMI ≥ 40 kg/m^2^, or 35 kg/m^2^ with the presence of weight-associated complications, is classified as morbid obesity (Heymsfield and Wadden, 2017).

Excessive fat expansion during obesity leads to a chronic inflammatory state, which may cause damage to endogenous repair systems. Mesenchymal stromal/stem cells (MSC) are endogenous, self-renewing cells capable of differentiating into mature cell lineages, and are abundantly found in subcutaneous adipose and other tissues (Eirin et al., 2014, 2016, 2017). This cell population is endowed with an ability to attenuate immune responses, decrease fibrosis, and stimulate angiogenesis through paracrine activities (Bernardo et al., 2009; Reinders et al., 2010). Studies have demonstrated promising capabilities for tissue repair when MSC are expanded *in vitro* and exogenously transplanted into injured organs (Ebrahimi et al., 2013; Eirin et al., 2015; Saad et al., 2017). Being immunomodulatory and anti-inflammatory, MSC are a choice cell type for cell-based therapy.

However, in pathological conditions such as obesity, adipose tissue-derived MSC may potentially exhibit limited regenerative capacity to repair injured tissues because of altered cellular properties and functions, including the ability to proliferate or migrate toward injury sites. Such impairments might not only interfere with the endogenous repair capacity of tissues and blood vessels in obese individuals, but could also impede the feasibility of using autologous MSC for exogenous transplantation if needed to combat organ damage. In particular, in obesity the adipose tissue microenvironment might constitute an important instigator of MSC dysfunction, because obese adipose tissue develops a pro-inflammatory profile and harbors an increased burden of senescent cells (Xu et al., 2015).

Cellular senescence is a stress response mechanism, which leads to irreversible, cell-cycle arrest mediated by the tumor suppressor proteins p53, p21, p16^INK4A^, and p19^ARF^ (Childs et al., 2016; Sturmlechner et al., 2017). Moreover, senescent cells release diverse growth factors, cytokines, chemokines, and matrix metalloproteinases, which comprise the senescence-associated secretory phenotype (SASP) (Baker et al., 2011). Senescent and SASP factors determine the fate of cells and their neighboring *milieu* by adversely affecting the microenvironment (Baker et al., 2011; Xu et al., 2015; Schafer et al., 2017).

Our lab has demonstrated that, in addition to inducing kidney (Ma et al., 2016) and cardiac (Zhang et al., 2015) damage, obesity impairs the function of adipose tissue-derived MSC in a large animal model (Zhu et al., 2016). Other findings have demonstrated that obese mice develop cellular senescence in their pre-adipocytes (Escande et al., 2014; Palmer et al., 2019). Hence, activation of injurious pathways may be compounded by diminished repair capacity. However, the effect of obesity on cellular senescence and on the reparative potential of MSC in human subjects remains obscure. Therefore, this study was designed to test the hypothesis that obesity induces cellular senescence and decreases functionality in MSC of obese subjects. To test our hypothesis, we compared function and cellular senescence in adipose tissue-derived MSC obtained from non-obese and obese subjects.

## Materials and Methods

### Subject Recruitment, Screening, and Enrollment

We examined the functional and senescent characteristics in adipose tissue-derived MSC isolated from non-obese and obese subjects. Recruitment for this study focused on non-obese and obese individuals evaluated at Mayo Clinic in Rochester, Minnesota in the Nephrology, Endocrine, and General Bariatric and Obesity clinics between October 2017 and March 2019. Eligible subjects were 18–80 years of age with a BMI ≤ 30 kg/m^2^ (non-obese) or BMI ≥ 30 kg/m^2^ (obese). The Mayo Clinic Institutional Review Board approved all experimental study procedures. Obese subjects scheduled for weight-reduction surgery and kidney donor candidates scheduled for nephrectomy, who gave written informed consent, underwent an adipose tissue (0.5–2.0 g) sampling at the time of their surgical procedures.

### Isolation and Culture of MSC

After harvesting, MSC were isolated from tissue specimens following standard protocols (Eirin et al., 2012; Saad et al., 2017). Briefly, minced adipose tissue was aseptically processed by incubation with collagenase-H at 37°C for 45 min. After digestion with collagenase-H, serum-containing medium was added to the suspension and filtered through a 100 μm cell strainer. The cellular suspension was centrifuged for 5 min at 1000 RPM to pull-down cells, and the cellular pellet re-suspended in Advanced Minimum Essential Medium supplemented with 5% platelet lysate (PLTmax, Mill Creek Life Sciences, Rochester, MN, United States). MSC were then expanded in culture for three passages to prepare for experimentation.

### MSC Phenotyping

Third-passage MSC were characterized by imaging flow cytometry (FlowSight, Amnis, Seattle, WA, United States) to confirm expression of MSC-specific surface markers (all from Abcam, San Francisco, CA, United States) CD73 (Cat.# ab106677), CD90 (Cat.# ab124527), and CD105 (Cat.# ab53321). Conversely, MSC were expected to not express CD45 (Cat.# ab51482) or CD14 (Cat.# ab82012). All antibodies were used at the manufacturer’s recommended dilutions and cellular concentrations, and data analyzed using Amnis^®^ Image Data Exploration and Analysis Software (IDEAS version 6.2) (Aghajani Nargesi et al., 2018).

Furthermore, MSC were characterized by their ability to differentiate into adipocyte, osteocyte, and chondrocyte lineages using a Human MSC Functional Identification Kit (R&D Systems^®^, Minneapolis, MN, United States, Cat.# SC006) (Zhu et al., 2016). Initially, MSC were seeded according to the manufacturer’s instructions, and medium changed every 3–4 days. Culture media contained specific supplements necessary to induce *trans-*differentiation into the appropriate mesenchymal lineage. Following 21 days of culture, mature cell phenotypes were detected by immunofluorescent staining using the provided antibodies: anti-mouse FABP4, anti-human Aggrecan, and anti-human Osteocalcin to distinguish adipocytes, chondrocytes, and osteocytes, respectively.

### MSC Functional Analyses

Cellular proliferation was evaluated using Incucyte^®^, a live-cell analysis and imaging system (Satorius, Ann Arbor, MI, United States). Approximately 2.5 × 10^3^ MSC/well were seeded in a 96-well plate and allowed to propagate in culture for 72 h. MSC migratory function was tested using a QCM^TM^ Colorimetric Cell Assay (EMD Millipore, Burlington, MA, United States; Cat.# ECM508), performed according to the company’s standard protocol.

### Senescence and SASP Markers

Total RNA was isolated from 0.5 and 1 × 10^6^ cells using mirVana^TM^ PARIS kit (ThermoFisher Scientific, Waltham, MA, United States; Cat.# AM1556), first strand cDNA produced by SuperScript^TM^ VILO^TM^ cDNA synthesis kit (ThermoFisher Scientific, Cat.# 11755050), and the ΔΔCt method used to assess gene expression levels in MSC from non-obese and obese subjects. Adipogenic markers were assessed using the primers for CCAAT/enhancer-binding protein-α (*C/EBP*α, Cat.# HS00269972) and peroxisome proliferator-activated receptor-γ (*PPAR*γ, Cat.# HS01115513). Cellular senescence was ascertained by expression of the cell-cycle arrest markers *p16* (Cat.# H00923894), *p21* (Cat.# HS00355782), and *p53* (Cat.# HS01034249), as well as the SASP markers, interleukin-6 (*IL-6*, Cat.# HS00174131), monocyte chemoattractant protein-1 (*MCP-1*, HS00234140) and galactosidase (gal)-beta-1 (*GLB1*, Cat.# HS01035168). Vascular endothelial growth factor (*VEGF*, Cat.# HS00900055) was also evaluated. All TaqMan primers were purchased from ThermoFisher Scientific and gene expression normalized to TATA-binding protein (*TBP*, Cat.# HS00427620).

The ratio of phosphorylated/total γ-H2AX expression was evaluated by Western blot to assess for DNA damage in MSC, and the activity of β-gal, a participant in cellular senescence (Dimri et al., 1995), using an assay (Enzo, Farmingdale, NY, United States; Cat.# ENZ-KIT 129).

### Co-culture of MSC and Senescent Endothelial Cells

To evaluate the reparative potency of MSC from non-obese and obese subjects, co-culture experiments were performed. Briefly, commercially available human umbilical vein endothelial cells (HUVEC, Cell Applications, San Diego, CA, United States; Cat.# 200K-05f) were grown in endothelial cell growth medium (EGM^TM^-Plus Endothelial Cell Growth Media-Plus Bulletkit^TM^ Medium, Lonza, Cohasset, MN, United States; Cat.# CC-5035), seeded at a density of 3.5 × 10^5^ cells/well in a transwell plate (VWR, Radnor, PA, United States, Cat.# 2944-076), and divided into four groups. Group 1 cells were cultured under normal conditions, while group 2–4 cells were co-incubated with tumor necrosis factor-α (TNF-α, 10 ng/mL) and transforming growth factor-β1 (TGF-β1, 5 ng/mL) for 3 days to induce cellular injury (Khan et al., 2017). This medium was then replaced with fresh growth medium. Groups 3 and 4 were also subsequently co-cultured with MSC from either non-obese or obese subjects, respectively (1.75 × 10^5^ cells/well insert) for another 24 h. Afterward all cells (*n* = 5–7/group) were lysed and prepared for qPCR analyses.

Additionally, the angiogenic potential of HUVEC was tested in Groups 1–4. Following treatment and co-culture procedures HUVEC were seeded onto a Matrigel^®^ matrix-coated plate (CORNING, Corning, NY, United States; Cat.# 354433) at a final concentration of 7 × 10^4^ cells/500 μL, incubated overnight in a 37°C, 5% CO_2_ humidified incubator. HUVEC were observed under a Zeiss Axio Observer inverted microscope for the formation of tube-like structures. The network of tubes was counted in five different fields of view for each group.

### *In vitro* Assays

Apoptotic signal was detected in MSC from non-obese and obese subjects using a Dead-End^TM^ Fluorimetric Terminal deoxynucleotidyl transferase dUTP nick end-labeling (TUNEL) assay (Promega, Madison, WI, United States; Cat.# G3250). In brief, 6.0 × 10^4^ MSC were prepared in 4-well chamber slides, assayed using the manufacturer’s directions and visualized by green fluorescence for fragmented DNA (incorporation of fluorescein-12-dUTP). The percentages of TUNEL-positive cells were quantified in 12 fields of view using the Cytation-5 Cell Imaging Reader.

Cellular oxidative stress was assessed by staining with dihydroethidium (DHE, ThermoFisher Scientific, Cat.# D11347) in MSC from non-obese and obese subjects. Fluorescence intensity was calculated using ImageJ software.

### Statistical Analysis

Distribution of the data was evaluated using the Shapiro-Wilk Test. Normally-distributed data were represented as mean ± standard deviation, and non-normal data as median and interquartile range. Comparisons among groups were performed using either a two-sample *t*-test with a 5% type-I error rate or Wilcoxon Rank Sum, as appropriate. One-way analysis of variance (ANOVA) and *post hoc* pairwise testing were employed to detect differences in co-culture experiments, whereas cellular proliferation data were analyzed using repeated ANOVA. Bivariate correlation analysis was used to determine the direction of association between BMI and MSC parameters, and multivariate linear regression performed in two models among the variables with *p* < 0.2 in the bivariate analysis with Collinearity Diagnostics. Variables with non-normal distribution were log-transformed before entering the multivariate linear regression models. All data were considered significant if *p* ≤ 0.05. Statistical analysis was accomplished using JMP 14.1 Software.

## Results

A total of 40 subjects undergoing kidney donation or bariatric surgery donated adipose tissue for MSC isolation. Females accounted for >60% of participants, and mean age was 59 years. As expected mean BMI was significantly higher in obese subjects in comparison to non-obese (Table 1; *p* < 0.001). Co-morbidities such as hypertension, dyslipidemia, obstructive sleep apnea, and diabetes were also prevalent. Table 1 summarizes the demographics, clinical characteristics, and medication use of each group.

**TABLE 1:** Baseline characteristics of non-obese and obese subject cohorts (*n* = 40).

Parameter	Non-obese	Obese
**Demographics:**		
Number of Subjects	11	29
Female Sex	64%	69%
Caucasian Race	100%	100%
Age, Years	61 ± 6	57 ± 10
**Clinical:**		
Body mass index, kg/m^2^	25.7 ± 2.0	40.0 ± 8.5***
Hypertension	18%	41%
Dyslipidemia	27%	41%
Obstructive Sleep Apnea	0%	46%**
Asthma	0%	14%
Fatty Liver Disease	0%	10%
Glucose Intolerance/Diabetes	18%	45%
Gastro-esophageal Reflux	18%	31%
Depression	9%	48%*
**Medications:**		
Anti-hypertensive	18%	34%
Anticoagulation	9%	10%
Statins	18%	24%
Hypoglycemic	0%	21%
Antidepressants/Anti-Anxiety	9%	55%

*p ≤ 0.05, **p ≤ 0.01, ***p ≤ 0.001 (vs. non-obese).

**FIGURE 1:**
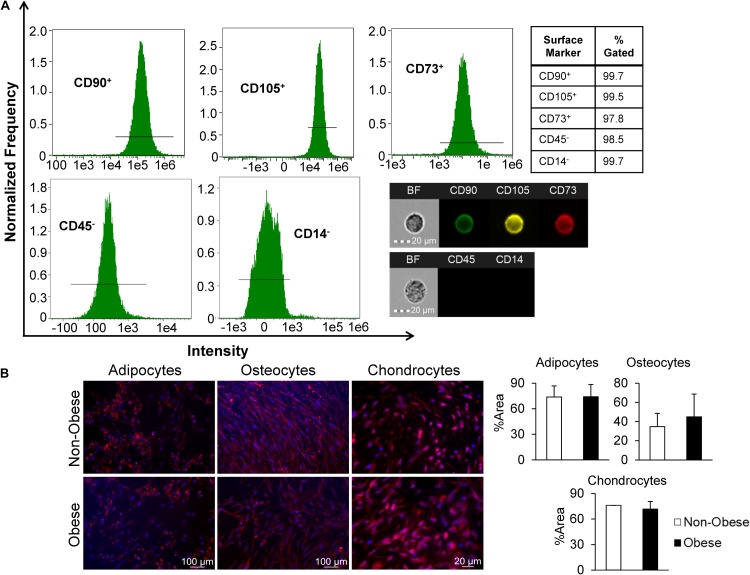
MSC derived from human adipose tissue exhibit attributes distinct to stem cells. **(A)** Markers of MSC were measured by imaging flow cytometry, which defined MSC as CD90^+^, CD105^+^, CD73^+^, and CD45,^–^ and CD14^–^, as shown in representative histograms and single-cell images. **(B)** MSC from non-obese and obese subjects demonstrated similar trilineage differentiation potential *in vitro*. Immunofluorescent staining (red; DAPI-blue stained nuclei) illustrates that similar expression of FABP4 (adipocytes), Osteocalcin (osteocytes), and Aggrecan (chondrocytes) in MSC from non-obese and obese subjects.

Mesenchymal stromal/stem cells-specific cell surface markers studied in passage-3 adipose tissue-derived MSC using flow cytometry demonstrated robust expression, with 99.7, 99.5, and 97.8% of the cell population positive for CD90, CD73, and CD105, respectively, whereas expression of both CD45 and CD14 was low (Figure 1A). For additional characterization, MSC were cultured in specific media to induce trilineage differentiation. After 21 days, we found that MSC from non-obese and obese subjects had similar abilities to *trans-*differentiate into adipocytes, osteocytes, and chondrocytes (Figure 1B). Altogether, these findings confirm characteristics indicative of MSC.

**FIGURE 2:**
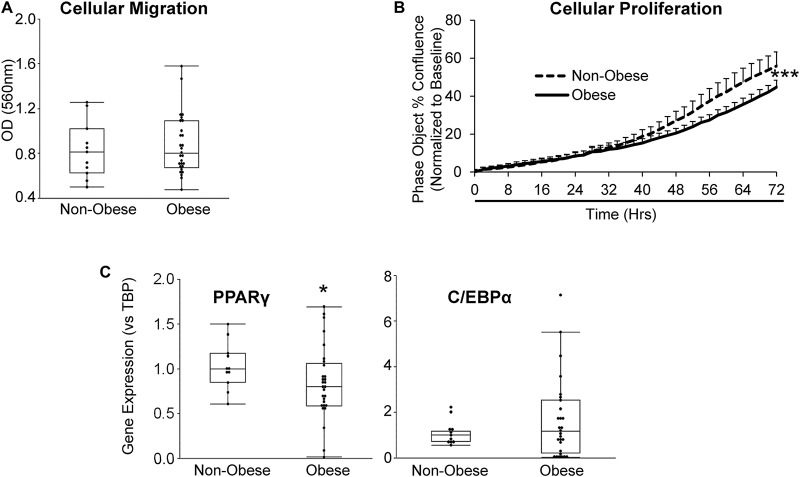
MSC from obese subjects demonstrate functional impairments. MSC from obese subjects showed intact cellular migration **(A)**, but lower proliferation **(B)** and expression of the adipogenic marker *PPAR*γ, but not *C/EBP*α **(C)** compared to MSC from non-obese subjects. *p ≤ 0.05, ***p ≤ 0.005 vs. non-obese.

Furthermore, we sought to evaluate whether obesity influences the migratory and proliferative capacities of MSC. Migration potential of MSC from obese subjects was comparable to MSC from non-obese subjects (Figure 2A), whereas MSC from obese subjects showed a significant reduction in proliferative activity starting at ∼32 h of incubation (Figure 2B). Gene expression of the adipogenic transcription factor *PPAR*γ was significantly lower in MSC from obese subjects, suggesting attenuated capacity for adipogenesis, while *C/EBP*α expression levels were similar between MSC from non-obese and obese subjects (Figure 2C).

**FIGURE 3:**
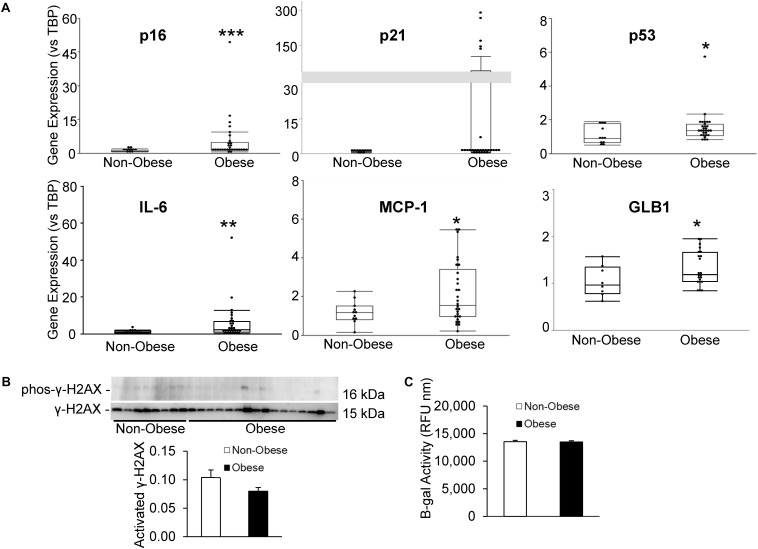
Human obesity evokes premature cellular senescence. Expression of senescence-associated and SASP genes **(A)** was upregulated in MSC from obese subjects. **(B)** γ-H2AX protein expression and **(C)** β-gal activity were unaltered in our obese cohort, although *GLB1* gene expression was upregulated. *p ≤ 0.05, **p ≤ 0.01, ***p ≤ 0.005 vs. non-obese.

Senescent cells were identified by relative expression of senescent and canonical SASP factors using qPCR. As shown in Figure 3A, MSC from obese subjects had markedly increased expression of *p16* and *p53*, as well as inflammatory SASP factors, *IL-6* and *MCP-1*, consistent with development of senescence. Additionally, *p21* tended to be elevated in MSC from obese subjects (*p* = 0.08, Figure 3A). Contrarily, in our obese subject population, there was no evidence of DNA damage, reflected in unaltered H2AX protein expression (Figure 3B). While β-gal enzyme activity was unaltered (Figure 3C), *GLB1* gene expression was upregulated.

**FIGURE 4:**
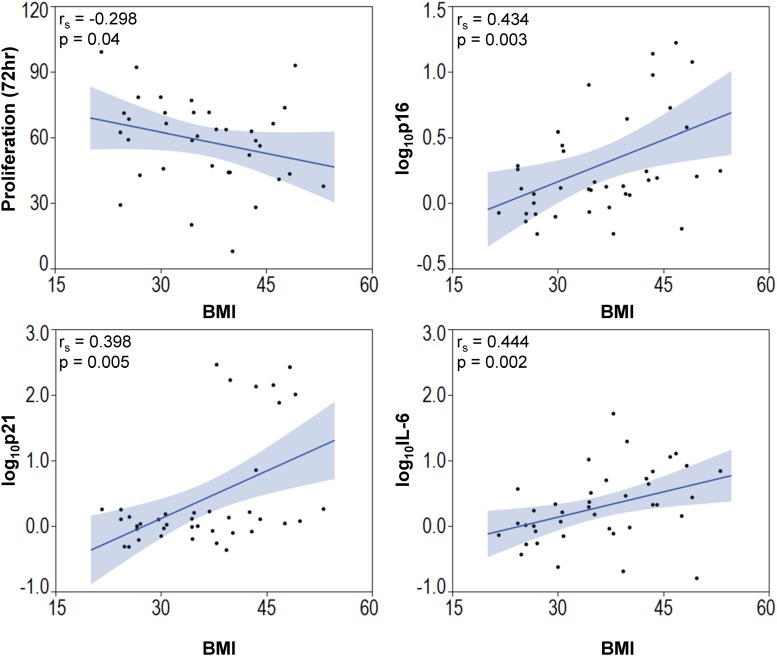
Body mass index is associated with elevated senescence-associated gene expression and decreased MSC proliferation. BMI of subjects correlated inversely with MSC proliferation and *p16*, *p21*, and *IL-6*.

Among the entire cohort, BMI correlated directly but modestly with the expression of the cellular senescence-related genes *p16* (Spearman’s correlation [r_s_] 0.434; *p* = 0.003) and *p21* (*r*_s_ = 0.398; *p* = 0.005), and with the SASP-related gene *IL-6* (*r*_s_ = 0.444; *p* = 0.002), and inversely with MSC proliferation (*r*_s_ = −0.298; *p* = 0.04, Figure 4). Multivariate analysis detected *p21* as the strongest predictor of obesity (Table 2, Model A). However, the presence of other predictors in Model B yielded *IL-*6 as the most significant correlate of BMI.

**TABLE 2:** Multivariate linear regression models for predictor variables of BMI (*n* = 40).

	Model A			Model B	
	Regression Coefficient (β)	*p*-value		Regression Coefficient (β)	*p*-value

Proliferation (72hr)	−0.205	0.164	Proliferation (72hr)	−0.148	0.310
Log-p16	0.149	0.489	Log-p16	0.241	0.132
**Log-p21**	**0.536**	0.001***	Log-p21	0.264	0.172
Log-p53	0.159	0.311	Log-p53	0.205	0.157
PPARγ	−0.106	0.479	PPARγ	0.025	0.871
Log-MCP-1	0.125	0.396	**Log-IL-6**	**0.578**	<0.001***

***p ≤ 0.001 Forward Selection. PPARγ, peroxisome proliferator-activated receptor-γ; MCP, macrophage chemoattractant protein-1; IL-6, interleukin-6. Values in bold font represent the strongest correlates for the model.

**FIGURE 5:**
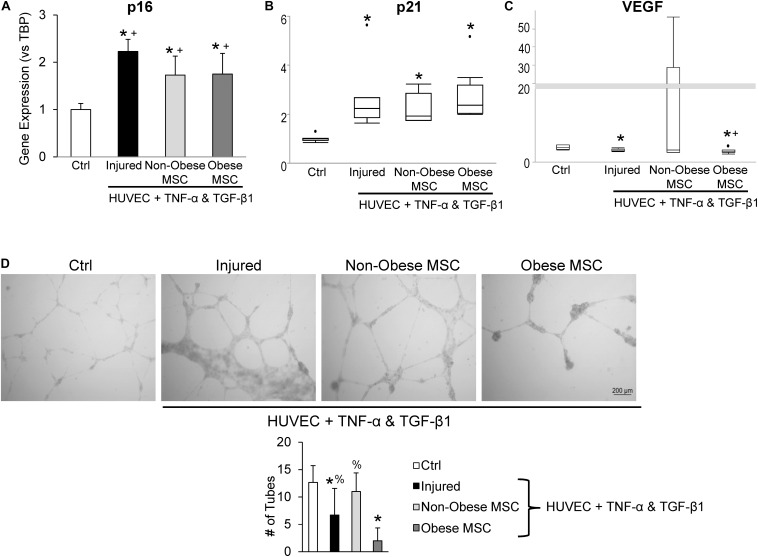
Co-culture with MSC from obese subjects fails to restore angiogenic potential of HUVEC. Gene expression of *p16* **(A)** and *p21* **(B)** increased and *VEGF* decreased **(C)** in injured HUVEC. Co-culture of HUVEC with both types of MSC attenuated HUVEC senescence, but only MSC from non-obese subjects restored *VEGF* expression. *In vitro* tube formation analysis shows evident tube-like structures in control (Ctrl) cells and non-obese MSC, whereas MSC from obese subjects fail to repair angiogenesis, and injure further **(D)**. *vs. ctrl, + vs. injured HUVEC, % vs. obese.

Additionally, we examined the repair capacity of MSC from non-obese and obese subjects on cellular injury and senescence *in vitro*. Pre-incubation with TNF-α and TGF-β1 generated cell-cycle arrest in HUVEC, suggested by upregulation of *p16* and *p21* gene expression (Figures 5A,B). Co-culture of injured HUVEC with both MSC from non-obese and obese subjects reduced expression of *p16* (Figure 5A), and MSC from non-obese subjects tended to attenuate expression of *p21* as well (Figure 5B, *p* = 0.08 vs. MSC from obese subjects). Furthermore, *VEGF* mRNA levels were significantly decreased in injured HUVEC, and increased by co-incubation with MSC from non-obese, but not with MSC from obese subjects (Figure 5C), suggesting that only the former repaired HUVEC. Tube formation analysis demonstrated that co-culture of injured HUVEC with MSC from non-obese subjects formed tube-like networks (Figure 5D), reflecting their pro-angiogenic properties. This activity was diminished in injured HUVEC both untreated and those co-incubated with MSC from obese subjects, detected by reduced tube numbers.

**FIGURE 6:**
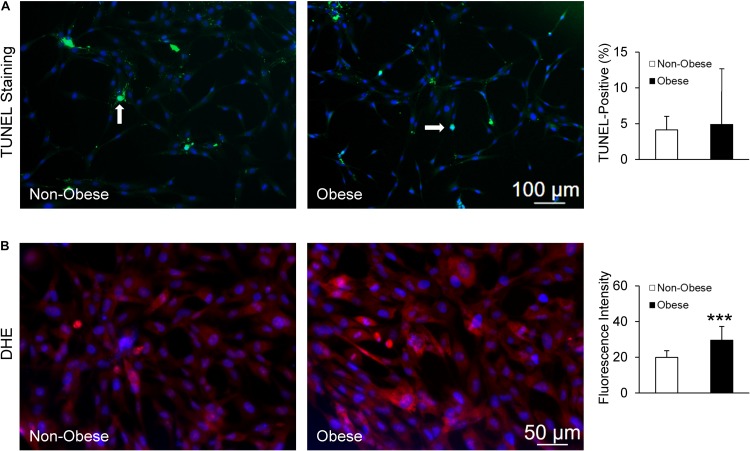
MSC from non-obese and obese subjects displayed similarly low apoptotic signals; white arrows indicate apoptotic nuclei (green), counterstained with DAPI (blue) **(A)**. Production of superoxide anion, DHE was increased in MSC from obese subjects **(B)**.

Finally, TUNEL staining showed no difference in apoptotic signals between MSC from non-obese and obese subjects (Figure 6A), whereas MSC from obese subjects exhibited markedly higher production of superoxide (Figure 6B).

## Discussion

This study demonstrates that human obesity provokes senescence and diminishes functionality in adipose tissue-derived MSC in comparison to MSC from non-obese controls, suggested by reduced MSC proliferation, expression of the differentiation-dependent factor *PPAR*γ, and pro-angiogenic efficacy. Furthermore, amplified expression of senescence-linked genes is directly associated with BMI. Taken together, these findings signify that the obesity microenvironment may limit the vitality and potency of MSC, which may account for increased propensity for tissue damage and organ injury observed in obesity.

In obesity, adipose tissue storage surpasses the given capacity and prompts disproportionate fat accumulation, leading to prolonged and uncontrolled inflammatory responses and disease progression. Dysfunction of adipose tissue is considered the main driver linked to the onset and progression of obesity-associated health problems, including insulin resistance, diabetes mellitus, hypertension, and dyslipidemia. Obesity is dramatically increasing worldwide, and if the trend continues, these health complications will also escalate (Hales et al., 2018).

Regenerative cell-based treatment strategies using adipose tissue-derived MSC are being extensively evaluated in various acute and chronic conditions. However, subject-associated comorbidities such as obesity may alter the biologic characteristics of MSC, compromising their efficacy for transplantation. We have previously shown that obesity also alters the function of adipose tissue-derived MSC and modifies the expression of genes associated with insulin signaling and mitochondrial function in an experimental pig model (Zhu et al., 2016; Conley et al., 2018; Meng et al., 2018a, b). In addition, obesity reduces the pro-angiogenic potential and “stemness” of MSC isolated from obese human subjects and their extracellular vesicles (Onate et al., 2012, 2013; Togliatto et al., 2016). Our study suggests that human obesity also induces cellular senescence, SASP, and impaired proliferation in MSC, each of which is linked to the severity of obesity.

Cellular senescence is implicated in the pathogenesis of several disease states, including obesity (Palmer et al., 2019), in which accumulation of senescent cells contributes to sterile inflammation. We identified increased expression of genes associated with cell-cycle arrest (*p16* and *p53*) and SASP (*IL-6* and *MCP-1*) in MSC isolated from obese compared to non-obese subjects, potentially contributing to the impaired cellular proliferation observed in MSC from obese subjects and amplifying senescent cell accumulation *in vitro* during culture expansion. Senescent cells accumulate in diseased tissues, and disrupt their structure and function by secreting a cocktail of factors known as the SASP (Baker et al., 2011). Xu et al. (2017) demonstrated that a small number of senescent cells can produce an osteoarthritic disease phenotype. Therefore, the presence of endogenous senescent MSC in obese subjects or their exogenous transplantation may further injure neighboring cells, including healthy MSC that are meant to repair, and accelerate a forward-feeding loop of intensified cellular senescence. Indeed, both MCP-1 (Jin et al., 2016) and IL-6 (Groppo and Richter, 2011) reinforce senescence. Upregulation of the senescence-associated (SA-β-gal) enzyme, an index of increased lysosomal activity, is a common marker for cellular senescence, and its activation is dependent on *GLB1*, the gene that encodes lysosomal β-gal (Lee et al., 2006). While our cohort showed no difference in β-gal activity between MSC from non-obese and obese subjects, *GLB1* expression was significantly increased, suggesting that the involvement of SA-β-gal might be at the gene expression level rather than enzymatic activity.

*C/EBP*α and *PPAR*γ are important regulators of adipogenic metabolism and development of functional fat progenitor cells. Blunted expression of these transcriptional factors can impair adipogenesis and insulin sensitivity (Dubois et al., 2006). Matulewicz et al. (2017) demonstrated that in a young cohort of overweight and obese individuals, adipose tissue expression of adipogenic markers significantly decreased in comparison to normal-weight individuals. Similarly, in our slightly older subject population we observed reduced expression of *PPAR*γ in adipose tissue-derived MSC, suggesting that MSC share impairments observed in surrounding adipose tissue. Moreover, Mitterberger et al. (2014) demonstrated that adipose-derived progenitor cells show reduced adipogenic capacity as senescent cells increase. Nevertheless, the tri-lineage differentiation assay indicated intact multi-lineage potential toward adipocytes, possibly due to culture conditions and unaltered expression of *C/EBP*α in the MSC. Our cohort also exhibited no measurable differences in γ-H2AX activation, arguing against DNA damage in their MSC at this early phase of morbidity.

Previous studies have shown that co-incubation of HUVEC with TNF-α and TGF-β1 induces cellular senescence and injury (Khan et al., 2017). Indeed, in our co-culture studies, gene expression of *p16* and *p21* was upregulated. Interestingly, MSC from both obese and non-obese subjects decreased expression of *p16*, whereas *p21* remained unchanged. In addition, VEGF, an important mediator of the pro-angiogenic function of endothelial cells (Cheng et al., 2019), and its expression falls in injured HUVEC (Wang et al., 2019; Wu et al., 2019), as we observed in our injured HUVEC model. Importantly, HUVEC *VEGF* gene expression was restored to control levels by co-incubation with MSC from non-obese subjects, whereas MSC from obese subjects failed to reinstate it. Moreover, MSC from non-obese subjects were able to stimulate angiogenesis activity in injured HUVEC, illustrated by formation of tube-like networks, whereas these capacities were attenuated in MSC from obese subjects. These observations imply that human adipose tissue-derived MSC have modest capability to blunt cellular senescence *in vitro*, which is unaffected by obesity. Contrarily, obesity significantly interferes with the potency of MSC to repair injured endothelial cells.

Additionally, we observed elevated superoxide levels in MSC from obese subjects. Inflammation and lipid peroxidation in obesity may increase oxidative stress, which in turn has been shown to induce senescence through p53-specific signaling (Han et al., 2020). However, additional studies need to be done conducted to further evaluate this potential mechanism.

The present study has limitations. While the relatively old age of our subjects is pertinent to the obese population (Jura and Kozak, 2016), the entirely Caucasian composition of our study cohort limits generalizability of our findings. Additionally, given the cohort sample size, we are unable to determine the dependence of obesity-induced MSC dysfunction on specific comorbidities. Nonetheless, this multi-morbidity prevalence is characteristic of the obese population. Furthermore, future studies need to define whether the MSC phenotype can be altered to disrupt the trajectory of obesity-induced MSC dysfunction and senescence. For example, we have recently demonstrated the effectiveness of senolytic agents in reducing senescence in kidneys of obese mice (Kim et al., 2019) and adipose tissue in subjects with diabetic kidney disease (Hickson et al., 2019). The ability of anti-inflammatory approaches to transiently interrupt the vicious cycle of the SASP and cellular senescence also requires further study.

## Conclusion

In summary, we show that human obesity alters functional characteristics and induces senescence in adipose tissue-derived MSC. This impairment in endogenous cellular repair systems may permit development and inadequate repair of lesions in subjects with obesity, and limit the utility of exogenous autologous delivery of their MSC. Further studies are needed to identify strategies to improve MSC function in obese individuals.

## Data Availability Statement

The raw data supporting the conclusions of this article will be made available by the authors, without undue reservation, to any qualified researcher.

## Ethics Statement

The studies involving human participants were reviewed and approved by Mayo Clinic Institutional Review Board. The patients/participants provided their written informed consent to participate in this study.

## Author Contributions

SC, LH, TTc, JK, and LL contributed conception and design of the study. SC, LH, HT, KJ, IS, JW, BI, MA, TTc, JK, and LL collected the data, and contributed to data analysis and interpretation. LH, TK, TM, JH, TTa, JK, and LL acquired the study sample and or research materials. SC, LH, JK, and LL provided financial support. SC wrote the first draft of the manuscript. All authors contributed to manuscript revision, read and approved the submitted version.

## Conflict of Interest

LL received grant funding from Novo Nordisk, and is an advisor to Weijian Technologies and AstraZeneca.

The remaining authors declare that the research was conducted in the absence of any commercial or financial relationships that could be construed as a potential conflict of interest.
